# Machine learning model for predicting out-of-hospital cardiac arrests using meteorological and chronological data

**DOI:** 10.1136/heartjnl-2020-318726

**Published:** 2021-05-17

**Authors:** Takahiro Nakashima, Soshiro Ogata, Teruo Noguchi, Yoshio Tahara, Daisuke Onozuka, Satoshi Kato, Yoshiki Yamagata, Sunao Kojima, Taku Iwami, Tetsuya Sakamoto, Ken Nagao, Hiroshi Nonogi, Satoshi Yasuda, Koji Iihara, Robert Neumar, Kunihiro Nishimura

**Affiliations:** 1 Department of Emergency Medicine, University of Michigan, Ann Arbor, Michigan, USA; 2 Department of Preventive Medicine and Epidemiologic Informatics, National Cerebral Cardiovascular Centre, Suita, Japan; 3 Department of Cardiovascular Medicine, National Cerebral and Cardiovascular Centre, Suita, Japan; 4 H.U. Group Research Institute G.K, Tokyo, Japan; 5 National Institute for Environmental Studies, Tsukuba, Japan; 6 Department of General Internal Medicine 3, Kawasaki Medical School, Kurashiki, Japan; 7 Health Service, Kyoto University, Kyoto, Japan; 8 Department of Emergency Medicine, Teikyo University, Itabashi-ku, Japan; 9 Cardiovascular Centre, Nihon University Hospital, Tokyo, Japan; 10 Shizuoka General Hospital, Shizuoka, Japan; 11 Department of Cardiovascular Medicine, Tohoku University Graduate School of Medicine, Sendai, Japan; 12 National Cerebral and Cardiovascular Center, Suita, Osaka, Japan

**Keywords:** cardiac arrest

## Abstract

**Objectives:**

To evaluate a predictive model for robust estimation of daily out-of-hospital cardiac arrest (OHCA) incidence using a suite of machine learning (ML) approaches and high-resolution meteorological and chronological data.

**Methods:**

In this population-based study, we combined an OHCA nationwide registry and high-resolution meteorological and chronological datasets from Japan. We developed a model to predict daily OHCA incidence with a training dataset for 2005–2013 using the eXtreme Gradient Boosting algorithm. A dataset for 2014–2015 was used to test the predictive model. The main outcome was the accuracy of the predictive model for the number of daily OHCA events, based on mean absolute error (MAE) and mean absolute percentage error (MAPE). In general, a model with MAPE less than 10% is considered highly accurate.

**Results:**

Among the 1 299 784 OHCA cases, 661 052 OHCA cases of cardiac origin (525 374 cases in the training dataset on which fourfold cross-validation was performed and 135 678 cases in the testing dataset) were included in the analysis. Compared with the ML models using meteorological or chronological variables alone, the ML model with combined meteorological and chronological variables had the highest predictive accuracy in the training (MAE 1.314 and MAPE 7.007%) and testing datasets (MAE 1.547 and MAPE 7.788%). Sunday, Monday, holiday, winter, low ambient temperature and large interday or intraday temperature difference were more strongly associated with OHCA incidence than other the meteorological and chronological variables.

**Conclusions:**

A ML predictive model using comprehensive daily meteorological and chronological data allows for highly precise estimates of OHCA incidence.

Out-of-hospital cardiac arrest (OHCA) is becoming a substantial worldwide health burden.^1 2^ A systematic review of the international epidemiology of OHCA from 1991 to 2007 reported that the estimated incidence of emergency medical services (EMS)-attended OHCA per 100 000 person-years was 86.4 in Europe, 98.1 in North America, 52.5 in Asia and 112.9 in Australia. The percentage of patients with survival to discharge is extremely low: 9.4% in Europe, 6.3% in North America, 2.2% in Asia and 10.7% in Australia.^2^ Accurately predicting the daily incidence of OHCA may provide a significant public benefit. Since the incidence of OHCA is affected by meteorological conditions,^3–10^ the application of high-resolution meteorological data to medicine might provide ways to improve predictions of the daily incidence of OHCA.

Machine learning (ML) has recently emerged as a novel approach to integrate multiple quantitative variables to improve diagnosis and accuracy of incidence predictions in cardiovascular medicine.^11–13^ Since meteorological data are very extensive and complex, ML can help identify associations not identified by conventional one-dimensional statistical approaches. By combining OHCA data with high-resolution meteorological data, such as daily forecasts, ML could use advanced analytics to build a warning system for individuals potentially at risk for OHCA of cardiac origin through internet of things (IoT) devices.

This study presents a predictive model for robust estimation of daily OHCA incidence of cardiac origin using a suite of ML approaches. This model was evaluated using a nationwide database of OHCA, as well as comprehensive meteorological data and chronological data.

## Methods

### Study design and setting

We matched two datasets between 1 January 2005 and 31 December 2015 at the hour level based on the time of the emergency call: the All-Japan Utstein Registry of the Fire and Disaster Management Agency (FDMA) dataset on patients with OHCA of cardiac origin and a meteorological dataset from the Weather Company, an IBM Business (Atlanta, Georgia, USA). We classified data from 1 January 2005 to 31 December 2013 in this merged dataset as the training dataset for developing the predictive models and data from 1 January 2014 to 31 December 2015 as the testing dataset for assessing whether the predictive models can work in other years. Japan has an area of approximately 378 000 km^2^ and its population was approximately 127 million in 2005.^14^ Online supplemental figure 1 shows three representative cities located at different latitudes in Japan: Sapporo at N43°, Kobe at N34° and Naha at N26°.

10.1136/heartjnl-2020-318726.supp1 Supplementary data

We performed a four-step analysis. First, we elucidated the association between the incidence of OHCA and daily meteorological and chronological variables. Second, we developed an ML predictive models for OHCA incidence based on daily meteorological data, chronological data and combined meteorological and chronological data from the training dataset.^15 16^ Third, we examined the concordance between the predicted incidence of OHCA based on the ML model and the observed incidence of OHCA in a testing dataset. To further examine concordance at the district level after the time period covered by the original dataset, we performed heatmap analysis using another dataset on the location of OHCA in Kobe city between 1 January 2016 and 31 December 2018. The Kobe Municipal Fire Department has detailed information about where OHCA events occurred in certain districts. The population of Kobe city is more than 1.5 million. Its age distribution (population pyramid) is similar to that of Japan overall. Finally, we investigated the relative strength of the associations between meteorological variables and the incidence of OHCA in each predictive model. The main outcome was the daily incidence of OHCA.

A subcommittee for resuscitation science in the Japanese Circulation Society was provided with registry data following the prescribed governmental procedures.

### OHCA dataset

Patients with OHCA of cardiac origin in the FDMA’s All-Japan Utstein Registry were included. The All-Japan Utstein Registry is a prospective, population-based, nationwide registry of patients who have had an OHCA event. Data were prospectively recorded using the internationally standardised Utstein template.^17^ The following patient information was collected and analysed: age, sex, aetiology of arrest (ie, cardiac or non-cardiac) and time of the emergency call. All event times were synchronised with the dispatch centre clock. In Japan, all patients with OHCA who received prehospital resuscitation efforts by EMS personnel are transported to a hospital because they are not permitted to terminate resuscitation in the field. Data were stored on the FDMA registry database server and checked for missing values. If a data form was incomplete, the FDMA returned it to the respective fire station for completion. The registry has yielded some findings about patients with OHCA.^14 18–21^ Details of the registry are described in the online supplemental materials.

### Meteorological and chronological dataset

We analysed meteorological data from the Weather Company (https://www.ibm.com/weather) that operates a weather forecasting service platform (online supplemental materials). Between 1 January 2005 and 31 December 2015, the resolution of the meteorological data was 30 km gridded points (Weather Company Data Packages). In 2016, the resolution improved to 4 km gridded points. Meteorological variables included ambient temperature (°C), relative humidity (%), precipitation during the previous hour (mm), snowfall (mm), cloud coverage (%), wind speed (kph) and atmospheric pressure (hPa). Chronological variables included year (2005 was considered year 0), season (spring: March–May; summer: June–August; autumn: September–November; winter: December–February, with winter coded as the reference value), day of the week (with Sunday coded as the reference value), holidays and Japanese holiday season from 29 December to 6 January (categorical variable with a value of 0 or 1).

### Development of predictive models

To develop predictive models for the daily incidence of OHCA, we used the the eXtreme Gradient Boosting (XGBoost) algorithm, which is an optimised distributed gradient boosting library widely used by data scientists for many ML challenges.^11–13^ Hyperparameters of the XGBoost algorithm were chosen to maximise the predictive ability of the model using fourfold cross-validation. In fourfold cross-validation, we classified our dataset into four groups, and the XGBoost algorithm fitted decision trees to three groups and used the remaining group for validation. This procedure was performed four times with a different validation group each time. Population size for each prefecture was included in the XGBoost algorithm as an offset term. We did not use ambient air pressure to avoid possible multicollinearity.

### Statistical analysis

The characteristics of present dataset were summarised with medians and IQRs for continuous variables and numbers and percentages for categorical variables by prefecture and day in the training and testing datasets. The generalised linear models (GLMs) based on the Poisson distribution investigated the associations between meteorological variables and daily OHCA incidence by prefecture using all data in univariable models and a multivariable model. We exponentiated regression coefficients and 95% CIs to present incidence rate ratios (IRRs) for estimated OHCA incidence with each 1-unit increase in a meteorological variable. A p value of less than 0.05 was considered to indicate a significant difference.

We evaluated the predictive accuracy of the predictive models based on mean absolute error (MAE) and mean absolute percentage error (MAPE) between predicted values calculated by the predictive models and observed daily OHCA incidence by prefecture. MAE reflects the average magnitude of differences between predicted values and observed values. MAE ranges from zero to infinity. Lower MAE values indicate higher predictive performance. MAPE is generally used as a measure of the predictive accuracy of a forecasting method. It is an average of the absolute values of errors divided by observed values. MAPE ranges from 0% to 100%. Lower MAPE values indicate higher model predictive performance. In general, MAPE less than 10% is considered highly accurate predicting.^22^ We also calculated correlation coefficients, which can range from −1.00 to 1.00. Higher absolute values indicate higher model predictive performance.

We investigated the relative strength of the associations between each meteorological variable and OHCA incidence in the ML predictive model using a Shapley Additive Explanations (SHAP) algorithm.^23^ For a given set of feature values, a SHAP value reflects how much a single variable, in the context of its interaction with other variables, contributes to the difference between the actual prediction and the mean prediction.

All statistical analyses were performed with R statistical software V.3.5.0 (https://www.R-project.org/) and the xgboost package for R V.0.71.2 (https://CRAN.R-project.org/package=xgboost). Full analysis dataset had no missing information in any key variables.

## Results

### Characteristics of the training and testing datasets

Among the 1 299 784 OHCA cases in the All-Japan Utstein Registry between 2005 and 2015, there were 661 052 OHCA cases of cardiac origin matched with meteorological data; 525 374 cases were in the training dataset on which fourfold cross-validation was performed, and 135 678 cases were in the testing dataset. The characteristics of the datasets are summarised in table 1. Between 2005 and 2015, the median daily incidence of OHCA increased from 133 (IQR 109–167) to 173 cases (IQR 146–216) and the median annual incidence of OHCA increased from 44.5 to 59.7 per 100 000 person-years. The median age of OHCA onset increased from 77 (IQR 66–85) to 80 years (IQR 70–87), and the proportion of males decreased from 59% to 57%. Various meteorological changes were observed in representative prefectures located at different latitudes over the study period. The median of the mean ambient temperature within a day increased from 5.6°C (IQR −2.8 to 14.8) to 7.1°C (IQR −1.5 to 14.6) in Sapporo at N43°. This trend was not observed in Kobe at N34° or Naha at N26°. Differences between maximum and minimum ambient temperatures within a day decreased from 7.0°C (4.1–10.2) to 6.4°C (4.1–9.2) in Sapporo, but increased from 5.0°C (3.5–6.4) to 5.4°C (4.0–6.9) in Kobe.

**Table 1:** Characteristics of daily data in the training (2005–2013) and testing (2014–2015) datasets

Variable	Training dataset (n=525 374)			Testing dataset (n=135 678)
	2005–2007	2008–2010	2011–2013	2014–2015
Demographic variables				
Daily incidence of OHCA, cases, median (IQR)	133 (109–167)	154 (127–187)	168 (138–212)	173 (146–216)
Incidence of OHCA, per 100 000 person-years	44.5	51.2	57.0	59.7
Patient characteristics				
Age, years, median (IQR)	77 (66–85)	79 (68–86)	80 (69–87)	80 (70–87)
Male sex, n (%)	89 872 (59)	101 276 (57)	110 336 (56)	76 892 (57)
Meteorological variables				
Ambient temperature, °C				
Mean value within a day				
Sapporo	5.6 (−2.8–14.8)	6.5 (−1.8–15)	6.5 (−2.8–15.4)	7.1 (−1.5–14.6)
Kobe	16.3 (8.8–22.9)	16.3 (9.4–22.8)	15.7 (7.9–22.6)	16.5 (8.7–21.8)
Naha	23.9 (20.4–27.9)	23.6 (20.3–28.0)	23.7 (19.9–27.6)	24.1 (20.2–28.0)
Differences between maximum and minimum values within a day				
Sapporo	7.0 (4.1–10.2)	6.6 (4.0–9.7)	6.2 (3.9–8.9)	6.4 (4.1–9.2)
Kobe	5.0 (3.5–6.4)	4.8 (3.5–6.2)	5.3 (4.0–6.8)	5.4 (4.0–6.9)
Naha	1.0 (0.6–1.8)	1.1 (0.6–1.8)	1.2 (0.7–1.9)	1.2 (0.7–1.9)
Difference in mean value from the previous day				
Sapporo	0.1 (−1.2–1.3)	0.1 (−1.2–1.4)	0.1 (−1.2–1.3)	0.1 (−1.1–1.3)
Kobe	0.1 (−0.9–1.0)	0.2 (−0.8–1.0)	0.1 (−0.8–1.0)	0.2 (−0.8–0.9)
Naha	0.0 (−0.5–0.6)	0.1 (−0.4–0.6)	0.1 (−0.4–0.6)	0.1 (−0.4–0.6)
Relative humidity, %				
Sapporo	91.7 (84.3–96.9)	90.6 (83.5–94.6)	91.6 (86.2–94.6)	89.7 (82.5–94.0)
Kobe	76.9 (715–82.0)	76.5 (71.0–82.6)	76.7 (71.0–83.2)	77.2 (71.5–83.9)
Naha	80.6 (75.3–84.0)	80.2 (74.6–84.0)	80.3 (74.6–84.2)	79.6 (73.3–84.1)
Precipitation during the previous hour, mm				
Sapporo	1.0 (0.0–3.0)	1.0 (0.0–3.0)	1.0 (0.0–3.0)	0.9 (0.0–2.9)
Kobe	0.0 (0.0–0.0)	0.0 (0.0–1.0)	0.0 (0.0–0.0)	0.0 (0.0–0.0)
Naha	0.0 (0.0–2.0)	0.0 (0.0–2.0)	0.0 (0.0–2.0)	0.0 (0.0–2.0)
Snowfall, mm *				
Sapporo	22.0 (9.0–34.0)	19.0 (9.0–34.0)	28.0 (14.0–42.0)	28.0 (9.0–44.0)
Kobe	0.0 (0.0–0.0)	0.0 (0.0–0.0)	0.0 (0.0–0.0)	0.0 (0.0–0.0)
Naha	0.0 (0.0–0.0)	0.0 (0.0–0.0)	0.0 (0.0–0.0)	0.0 (0.0–0.0)
Cloud coverage, %				
Sapporo	91.4 (68.3–98.8)	89.9 (64.6–98.7)	88.8 (66.3–97.5)	84.3 (54.2–96.5)
Kobe	59.9 (33.1–81.7)	59.7 (35.2–84.9)	64.5 (37.5–86.4)	63.5 (36.1–87.1)
Naha	67.8 (32.8–92.0)	67.8 (32.0–92.0)	75.2 (44.1–93.8)	69.6 (36.4–91.4)
Wind speed, kph				
Sapporo	11.0 (7.9–14.6)	11.4 (8.1–15.1)	11.8 (8.1–16.5)	11.8 (8.3–16.6)
Kobe	11.2 (8.5–15.9)	11.4 (8.4–16.3)	10.2 (7.2–115.2)	9.8 (7.2–14.3)
Naha	24.2 (18.1–32.1)	24.5 (18.5–31.5)	26.1 (19.5–33.2)	25.0 (19.0–31.6)
Atmospheric pressure, hPa				
Sapporo	977 (972–981)	978 (973–982)	977 (972–981)	977 (973–982)
Kobe	1010 (1005–1015)	1010 (1006–1014)	1008 (1003–1012)	1008 (1004–1013)
Naha	1014 (1010–1019)	1014 (1010–1018)	1013 (1009–1018)	1014 (1010–1019)

Continuous values are presented as medians (IQR).

*Snowfall values were calculated for the winter (December–February).

†See online supplemental eFigure 1 for more information.

OHCA, out-of-hospital cardiac arrest.

### Association between meteorological and chronological variables and OHCA incidence

Online supplemental figure 2 shows the incidence of OHCA by each meteorological variable. The association between OHCA incidence of cardiac origin and mean ambient temperature was U-shaped, meaning that the incidence of OHCA was lowest at approximately 25°C and higher at temperatures above and below.

Exponentiated regression coefficients (ie, IRRs) of the GLM are shown in table 2. In univariable models, conventional ambient temperature, relative humidity, precipitation during the previous hour, snowfall, cloud coverage and wind speed were statistically associated with OHCA incidence (p<0.05, respectively). In the multivariable model, similar statistical associations were observed, except for mean precipitation during the previous hour, mean snowfall and mean wind speed for each day.

**Table 2:** Incidence rate ratios (IRRs) obtained with a GLM based on the Poisson distribution for both datasets

Predictor	Univariable model		Multivariable model*	
	IRR (95% CI)	P value	IRR (95% CI)	P value
Meteorological variables				
Mean values within a day				
Ambient temperature (per 5°C)	0.872 (0.870 to 0.873)	<0.001	0.874 (0.872 to 0.875)	<0.001
Relative humidity (per 10%)	0.977 (0.975 to 0.980)	<0.001	0.985 (0.980 to 0.990)	<0.001
Precipitation during the previous hour (per 1 mm)	0.913 (0.907 to 0.918)	<0.001	0.986 (0.972 to 1.000)	0.053
Snowfall (per 1 mm)	1.088 (1.085 to 1.091)	<0.001	1.004 (0.998 to 1.010)	0.184
Cloud coverage (per 10%)	0.992 (0.991 to 0.993)	<0.001	0.99 (0.989 to 0.991)	<0.001
Wind speed (per 10 kph)	1.061 (1.057 to 1.065)	<0.001	1.000 (0.993 to 1.007)	0.984
Atmospheric pressure (per 1 hPa)	0.994 (0.993 to 0.995)	<0.001	NA†	
Differences between maximum and minimum values within a day				
Ambient temperature (per 5°C)	1.012 (1.009 to 1.016)	<0.001	0.982 (0.976 to 0.988)	<0.001
Relative humidity (per 10%)	0.996 (0.994 to 0.998)	<0.001	0.996 (0.993 to 1.000)	0.035
Precipitation during the previous hour (per 1 mm)	0.98 (0.979 to 0.981)	<0.001	1.006 (1.003 to 1.009)	<0.001
Snowfall (per 1 mm)	1.335 (1.325 to 1.345)	<0.001	1.004 (1.003 to 1.006)	<0.001
Cloud coverage (per 10%)	1.006 (1.005 to 1.006)	<0.001	1.003 (1.002 to 1.003)	<0.001
Wind speed (per 10 kph)	1.053 (1.050 to 1.057)	<0.001	1.013 (1.008 to 1.018)	<0.001
Atmospheric pressure (per 1 hPa)	1.175 (1.169 to 1.181)	<0.001	NA†	
Difference in mean value from the previous day				
Ambient temperature (per 5°C)	1.003 (0.997 to 1.010)	0.280	1.075 (1.068 to 1.082)	<0.001
Relative humidity (per 10%)	0.990 (0.987 to 0.992)	<0.001	1.006 (1.002 to 1.010)	0.004
Precipitation during the previous hour (per 1 mm)	0.987 (0.983 to 0.991)	<0.001	0.984 (0.977 to 0.990)	<0.001
Snowfall (per 1 mm)	0.994 (0.991 to 0.998)	0.002	0.993 (0.989 to 0.996)	<0.001
Cloud coverage (per 10%)	0.997 (0.997 to 0.998)	<0.001	1.004 (1.003 to 1.005)	<0.001
Wind speed (per 10 kph)	0.990 (0.985 to 0.994)	<0.001	1.007 (1.002 to 1.013)	0.010
Atmospheric pressure (per 1 hPa)	1.017 (1.012 to 1.022)	<0.001	NA†	
Chronological variables				
Year (per 3 years)^‡,§^	1.059 (1.056 to 1.062)	<0.001	1.059 (1.056 to 1.062)	<0.001
Year squared^§^	0.998 (0.998 to 0.998)	<0.001	0.998 (0.998 to 0.998)	<0.001
Japanese holiday	1.154 (1.141 to 1.166)	<0.001	1.099 (1.087 to 1.111)	<0.001
Day of the week				
Sunday	1 (Ref.)		1 (Ref.)	
Monday	0.98 (0.971 to 0.988)	<0.001	0.971 (0.963 to 0.980)	<0.001
Tuesday	0.904 (0.896 to 0.913)	<0.001	0.905 (0.897 to 0.913)	<0.001
Wednesday	0.879 (0.871 to 0.886)	<0.001	0.879 (0.871 to 0.887)	<0.001
Thursday	0.88 (0.872 to 0.888)	<0.001	0.879 (0.872 to 0.887)	<0.001
Friday	0.889 (0.881 to 0.897)	<0.001	0.888 (0.880 to 0.896)	<0.001
Saturday	0.909 (0.901 to 0.917)	<0.001	0.909 (0.901 to 0.917)	<0.001
Season				
Winter	1 (Ref.)		1 (Ref.)	
Spring	0.742 (0.737 to 0.747)	<0.001	0.875 (0.868 to 0.882)	<0.001
Summer	0.56 (0.556 to 0.564)	<0.001	0.892 (0.880 to 0.904)	<0.001
Autumn	0.67 (0.666 to 0.675)	<0.001	0.892 (0.883 to 0.901)	<0.001

IRRs are exponentiated regression coefficients corresponding to multiplicative terms for the estimated number of cardiac arrests for each 1-unit, 5-unit or 10-unit increase in a meteorological variable.

*In the multivariable model, exponentiated regression coefficients were adjusted for prefecture and all of the variables listed in this table.

†Atmospheric pressure was excluded from the multiple Poisson regression analysis because of high multicollinearity.

‡In the model, 2005 was considered year 0.

§In the univariable model, we included both year and year squared in the same model.

GLM, generalised linear model; IRR, incidence rate ratio; NA, not applicable.

### Predictive accuracy of the models

**Figure 1:**
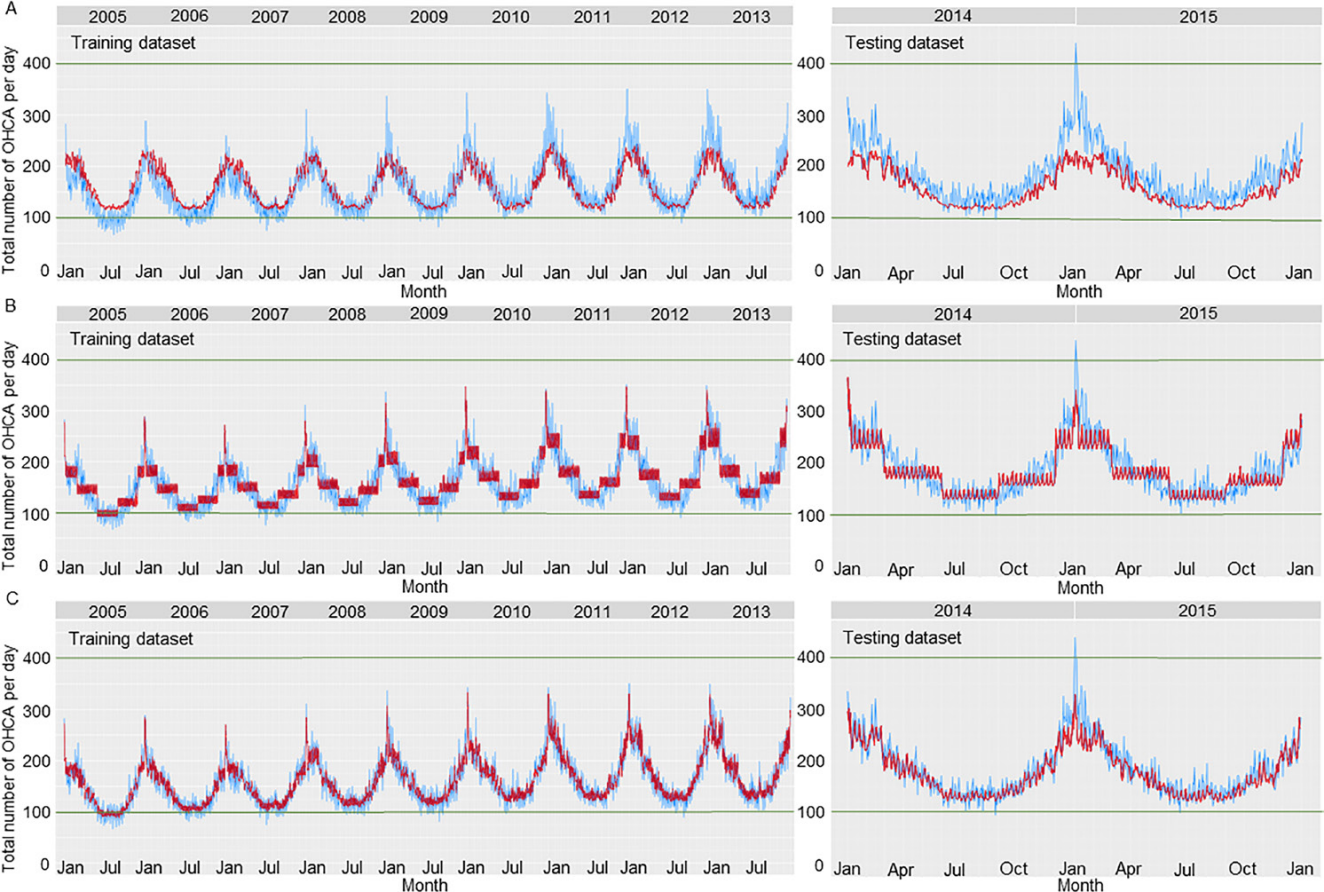
Observed versus predicted incidence of OHCA. The blue dots indicate the observed total number of OHCAs per day in Japan. The red dots indicate the predicted number based on the following predictive models: (A) ML model with comprehensive meteorological variables, (B) ML model with chronological variables and (C) ML model with combined meteorological and chronological variables. ML, machine learning; OHCA, out-of-hospital cardiac arrest.

Predicted and observed OHCA incidence of cardiac origin are plotted for each model in figure 1. Initially, we developed the predictive models based on comprehensive meteorological variables and chronological variables, respectively, using ML. The predicted values fitted the observed values well. ML predictive models with comprehensive meteorological variables were able to predict the daily change in OHCA incidence but were not able to predict a large increase in OHCA incidence accurately during the winter (figure 1A). ML predictive models with chronological variables were able to predict a large increase in OHCA incidence during the winter (figure 1B). By combining meteorological and chronological variables in a single ML predictive model, the concordance of the predicted values and the observed values improved (figure 1C). Predictive accuracy of the predictive models is shown in table 3. Among all predictive models, the predictive model with combined meteorological and chronological variables had the highest predictive accuracy in the training (MAE 1.314 and MAPE 7.007%) and testing datasets (MAE 1.547 and MAPE 7.788%). The predictive model with combined meteorological and chronological variables also had the highest correlations between observed and predicted values in the training (r=0.880, 95% CI 0.880 to 0.880) and testing datasets (r=0.870, 95% CI 0.860 to 0.870) (online supplemental figure 3).

**Table 3:** Accuracy of the predictive models for out-of-hospital cardiac arrest based on meteorological data, chronological data and combined meteorological and chronological data

Measure of predictive model performance	ML model with comprehensive meteorological variables		ML model with chronological variables		ML model with combined meteorological and chronological variables	
	Training dataset	Testing dataset	Training dataset	Testing dataset	Training dataset	Testing dataset
MAE by prefecture and day	1.413	1.628	1.415	1.577	1.314	1.547
MAPE by day (%)*	12.158	14.023	11.307	10.833	7.007	7.788

*In general, MAPE less than 10% is considered highly accurate predicting; 10%–20%: good predicting; 20%–50%: reasonable predicting; and more than 50%: inaccurate predicting.^22^

MAE, mean absolute error; MAPE, mean absolute percentage error; ML, machine learning.

**Figure 2:**
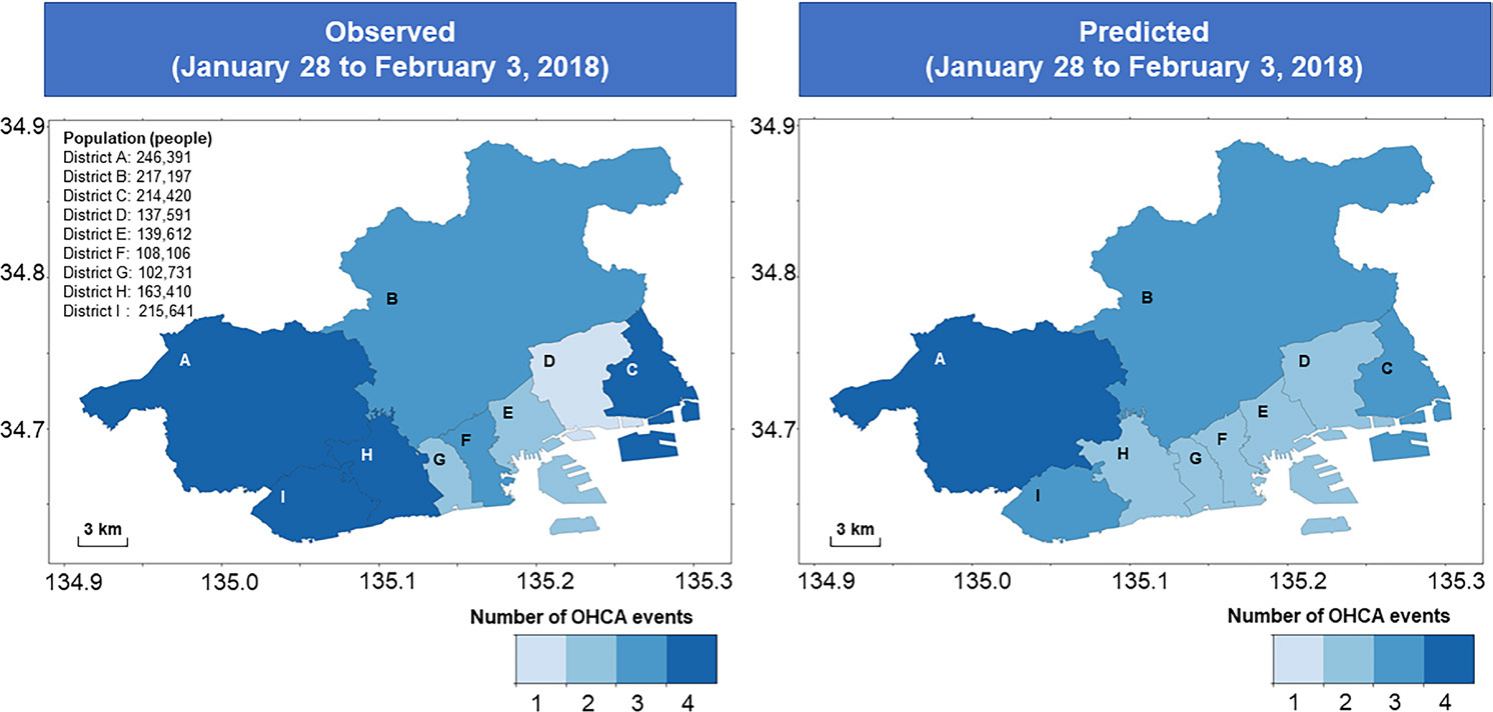
Heatmap of observed and predicted OHCA incidence at the district level in Kobe city during a 1-week period. The blue graduation indicates the number of OHCA events, ranging from 1 to 4. The population for each district is provided. OHCA, out-of-hospital cardiac arrest.

Moreover, using the predictive model with combined meteorological and chronological variables, we predicted the incidence of OHCA at a district level in Kobe city during a 1-week period after 2016. Figure 2 shows the heatmap of observed vs predicted numbers of OHCA incidence between 28 January and 3 February 2018. During this week, 24 OCHA events were predicted for Kobe city, while 27 OCHA events were observed. The heatmap showed that zero to four OHCA events occurred in each district during this week. Among nine districts, the predicted OHCA incidence matched the observed OHCA incidence in four districts (districts A, B, E and G). One fewer OHCA event was predicted than observed in three districts (districts C, F and I).

### Predictive importance

**Figure 3:**
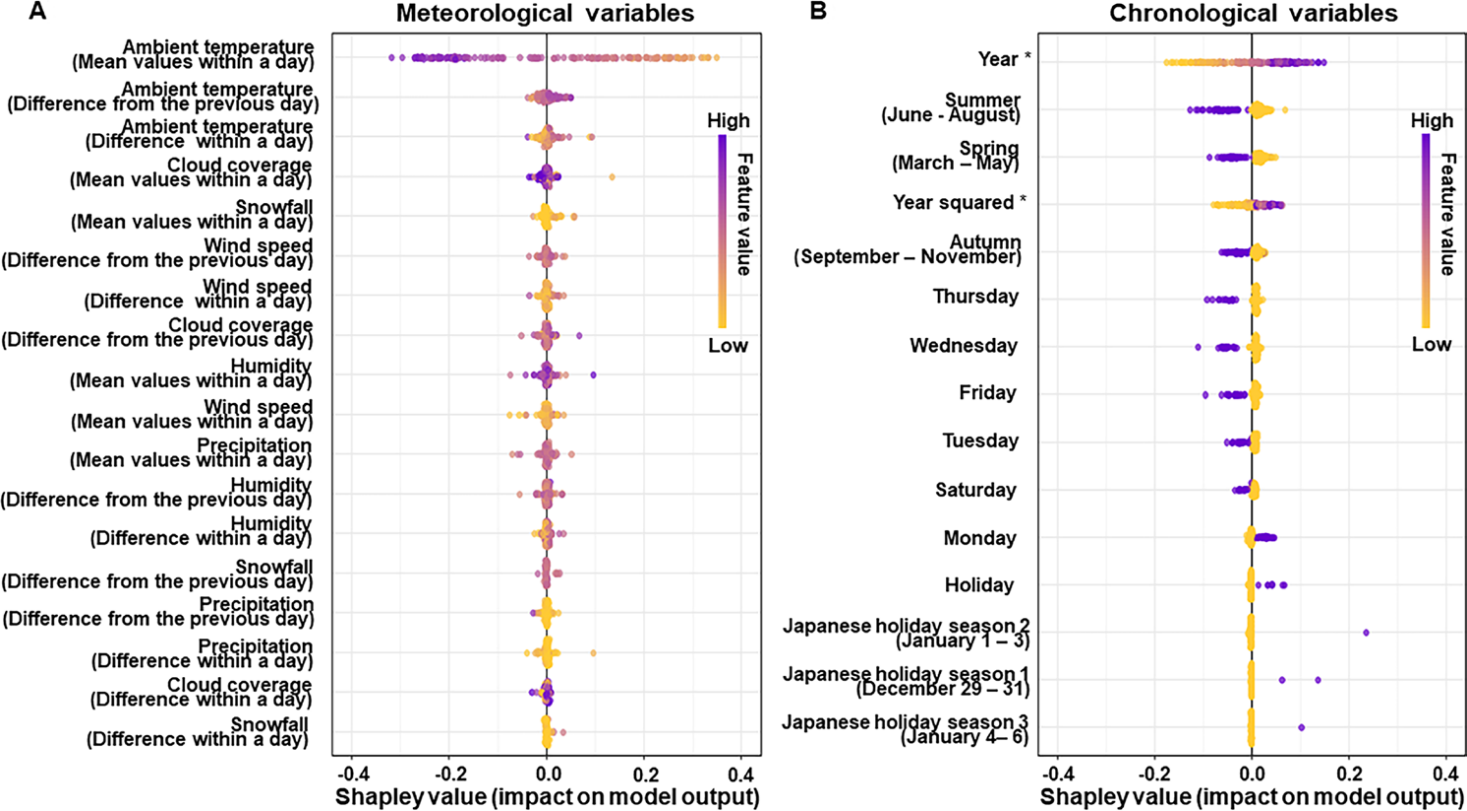
Importance of meteorological and chronological variables in a machine learning predictive model. This figure shows variable importance plots for meteorological (A) and chronological variables (B) in the machine learning predictive model using XGBoost. The yellow to purple dots in each row represent low to high values of the number of OHCA events corresponding to that meteorological or chronological variable. The x-axis shows the Shapley value, indicating the variable’s impact on the model. Positive SHAP values tend to drive predictions towards an OHCA event, and negative SHAP values tend to drive the prediction towards no OHCA events. *In the model, 2005 was considered year 0. OHCA, out-of-hospital cardiac arrest; SHAP, Shapley Additive Explanations; XGBoost, eXtreme Gradient Boosting.

The predictive importance of meteorological and chronological variables in the ML predictive model is shown in figure 3. With regard to meteorological variables, lower mean ambient temperature within a day was the most strongly associated with the incidence of OHCA. In addition, larger difference in mean ambient temperature from the previous day and larger difference between maximum and minimum ambient temperatures within a day were also more strongly associated with the incidence of OHCA than other variables. Among chronological variables, more recent year, winter, Sunday, Monday and holiday were more strongly associated with the incidence of OHCA.

## Discussion

In this study, using the predictive model developed with combined meteorological and chronological variables, we succeeded in predicting OHCA incidence of cardiac origin with high precision. Our study is the first to predict daily OHCA incidence based on both meteorological and chronological variables using ML.

Previous studies that investigated meteorological variables associated with the incidence of cardiovascular events used ambient temperature alone or seasons^8–10 24^ and were limited to one city or region.^8 9^ Thus, these studies did not take diversity in geography and climate into account. Indeed, Japan is located in a temperate zone with four distinct seasons and its climate varies from cool temperate in the north to subtropical in the south. Latitude ranges from N45° to N20°. In this study, we used ML to process complex data that included a nationwide registry of OHCA events and a comprehensive meteorological dataset. If climate change becomes more intense, the relationship between OHCA incidence and comprehensive meteorological data may become all the more crucial.

### Meaning of study

Our predictive model for the daily incidence of OHCA had high predictive accuracy. In particular, a larger difference in mean ambient temperature from the previous day and a larger range in ambient temperature within a day, in addition to mean ambient temperature lower or higher than 25°C within a day (a U-shaped distribution), were associated with OHCA incidence of cardiac origin. We speculated that a sudden change in ambient temperature on days with extreme cold or heat plays a key role in increasing the risk of OHCA of cardiac origin; this might be related to increased sympathetic tone and blood viscosity.^25 26^ However, in this study, we could not investigate how the location of OHCA affects the association between meteorological condition and the incidence of OHCA because detailed information about whether OHCA events occurred indoors or outdoors was not available. If this information is available in future research, one prevention method might be to advise individuals to stay home using an IoT device warning system on high-risk days. We found important chronological variables that affect the incidence of OHCA such as season, day of the week and holiday. Combining meteorological and chronological variables further improved the predictive accuracy of the ML predictive model. Importantly, at the local level, a heatmap showed that predicted OHCA incidence based on the ML predictive model fitted observed OHCA incidence well. Although our model was developed based on meteorological data with a resolution of 30 km gridded points, it could be applied even at a district level within one city. The model could be more practically useful if it could be further improved to predict OHCA incidence within a medical catchment area.

### Implications

One advantage of using meteorological data to make predictions of OHCA incidence is that weather forecasts can predict meteorological conditions 2 weeks ahead. Our predictive model for daily incidence of OHCA is widely generalisable for the general population in developed countries because this study had a large sample size and used comprehensive meteorological data. Many developed countries are located in a similar latitude range as Japan. The methods developed in this study serve as an example of a new model for predictive analytics that could be applied to other clinical outcomes of interest related to life-threatening acute cardiovascular disease. It could also provide more opportunities to support self-management in high-risk individuals through IoT devices.^27 28^ Moreover, we expect to use our predictive model to provide warnings to EMS personnel, in addition to citizens, on high-risk days. As a result, it may lead to shorter transport time from onset to hospital arrival and rapid start of advanced resuscitation care after hospital arrival. Future research should prospectively evaluate the effectiveness of this approach and whether it translates into improved clinical outcomes.

### Strengths and limitations

Our study has several strengths. First, the All-Japan Utstein Registry included all patients with OHCA who received prehospital resuscitation efforts by EMS personnel because they are not permitted to terminate resuscitation in the field. Moreover, uniform data collection, a large sample size and a population-based design covering all known OHCA events in Japan minimise potential sources of bias. These features contribute to the representativeness of the present predictive models.

This study has several inherent limitations. First, we did not have detailed information about where OHCA events occurred in various districts except in Kobe city; information was generally only available on the prefecture level. Second, our data did not address the potential variability in patients’ preexisting medical conditions. Third, the predictability of future OHCA events will depend on the accuracy of meteorological data. Finally, external testing in other developed countries was not performed.

## Conclusion

An ML predictive model using combined multiple meteorological and chronological variables could predict OHCA incidence of cardiac origin with high precision. Furthermore, this predictive model may be useful for preventing OHCA and improving the prognosis of patients with OHCA via a warning system for citizens and EMS on high-risk days in the future.

**Key messages**

**What is already known on this subject?**

- Previous studies have shown an association between lower ambient temperature and the incidence of cardiovascular events.

**What might this study add?**

- This study using Japanese out-of-hospital cardiac arrest (OHCA) registry data combined with high-resolution meteorological and chronological data demonstrated that various meteorological and chronological variables are significantly associated with the incidence of OHCA of cardiac origin. A machine learning predictive model developed with comprehensive meteorological and chronological variables predicted the daily incidence of OHCA with high precision. Sunday, Monday, holiday, winter, low ambient temperature, and large interday or intraday temperature difference were strongly associated with OHCA incidence.

**How might this impact on clinical practice?**

- This predictive model may be useful for preventing OHCA and improving the prognosis of patients with OHCA via a warning system for citizens and emergency medical services on high-risk days, thereby in the future.

## Data Availability

No data are available. The All-Japan Utstein Registry of the FDMA is a publicly accessible open database. The availability of the Kobe Municipal Fire Department database, which includes detailed information on the location of cardiac arrest used with permission for this study, is restricted. The Weather Company Data Packages from the Weather Company is subject to a full license agreement, which does not permit data sharing outside of the research team.
