# Supplementary material for: Machine learning model for predicting out-of-hospital cardiac arrests using meteorological and chronological data
Source: Heart. 2021 May 17;107(13):1084–91. doi: 10.1136/heartjnl-2020-318726 (PMC8223656; doi:10.1136/heartjnl-2020-318726)
Supplement: Supplementary data [file heartjnl-2020-318726supp001.pdf]

SUPPLEMENTARY MATERIAL

TABLE OF CONTENTS

1. Out-of-hospital cardiac arrest dataset **2**

2. Meteorological dataset **4**

3. Supplemental Figures **5**

Supplementary Figure 1. Latitude range in Japan **5**

Supplementary Figure 2. Incidence of out-of-hospital cardiac arrest by meteorological condition **6**

Supplementary Figure 3. Correlations between observed and predicted numbers of out-of-hospital cardiac arrests **7**

4. References **8**

Out-of-hospital cardiac arrest dataset

Patients with out-of-hospital cardiac arrest (OHCA) of cardiac origin in the Fire and Disaster Management Agency (FDMA) All-Japan Utstein Registry were included. The All-Japan Utstein Registry is a prospective, population-based, nationwide registry of patients who have had an OHCA event. Data were prospectively recorded using the internationally standardized Utstein template.[1] Details of the registry have been described previously. The registry has yielded some findings about patients with OHCA.[2-8]

In Japan, there are 802 fire stations governed by municipalities with dispatch centers operating 24 hours each day. All collaborating medical institutions participate in the registry. Data were collected prospectively by the FDMA starting from January 1, 2005, according to the Utstein guidelines. The following patient information was collected and analyzed: etiology of arrest (i.e., cardiac or noncardiac), sex, age, whether the arrest was witnessed by a bystander, relationship of the bystander to the patient (i.e., family member or other), first documented cardiac rhythm, type of bystander-initiated cardiopulmonary resuscitation (CPR) (i.e., CPR with chest compressions only or CPR with chest compressions and assisted breathing), whether CPR was assisted by an emergency medical system (EMS) dispatcher, whether public-access defibrillation was delivered by a bystander, and return of spontaneous circulation status before hospital arrival. The resuscitation time-course variables included time of emergency call placement, initiation of CPR, defibrillation, epinephrine administration, prehospital return of spontaneous circulation (ROSC), and hospital arrival. The FDMA established the registry cohort, collected the data, and provided data quality assurance. All event times were synchronized with the dispatch center clock. Data were stored on the FDMA registry database server and checked for missing or repeated data by the computer system using an Utstein-style online statistical survey system. If a data form was incomplete, the FDMA returned it to the respective fire

station for completion. In Japan, all patients with OHCA who receive prehospital resuscitation efforts by EMS personnel are transported to a hospital because they are not permitted to terminate resuscitation in the field. Cardiac arrest was defined as the cessation of cardiac mechanical activity, as confirmed by the absence of any signs of circulation.

To further examine the concordance between the incidence of OHCA predicted by the machine learning model and the actual incidence of OHCA at the district level in Kobe city after the time period in the original dataset, we obtained data on the location of OHCA in Kobe city between 2016 and 2018 from the Kobe Municipal Fire Department.

Nakashima T, *et al. Heart* 2021; 107:1084–1091. doi: 10.1136/heartjnl-2020-318726

Meteorological dataset

We analyzed meteorological data from the Weather Company, an IBM business (https://www.ibm.com/weather) that provides a weather forecasting service platform. The company provides approximately 26 billion weather forecasts for 3 billion points in the world every day. Forecasts are updated every 15 minutes with data from weather observation stations, radar, satellites, and internet of things devices like smartphones. Between 2005 and 2015, the meteorological data was at a resolution of 30-km gridded points (Weather Company Data Packages). In 2016, the resolution improved to 4-km gridded points. Meteorological data included ambient temperature (°C), relative humidity (%), precipitation during the previous hour (mm), snowfall (mm), cloud coverage (%), wind speed (kph), and atmospheric pressure (hPa).

Nakashima T, *et al. Heart* 2021; 107:1084–1091. doi: 10.1136/heartjnl-2020-318726

Supplementary Figure 1. Latitude range in Japan and districts in Kobe city

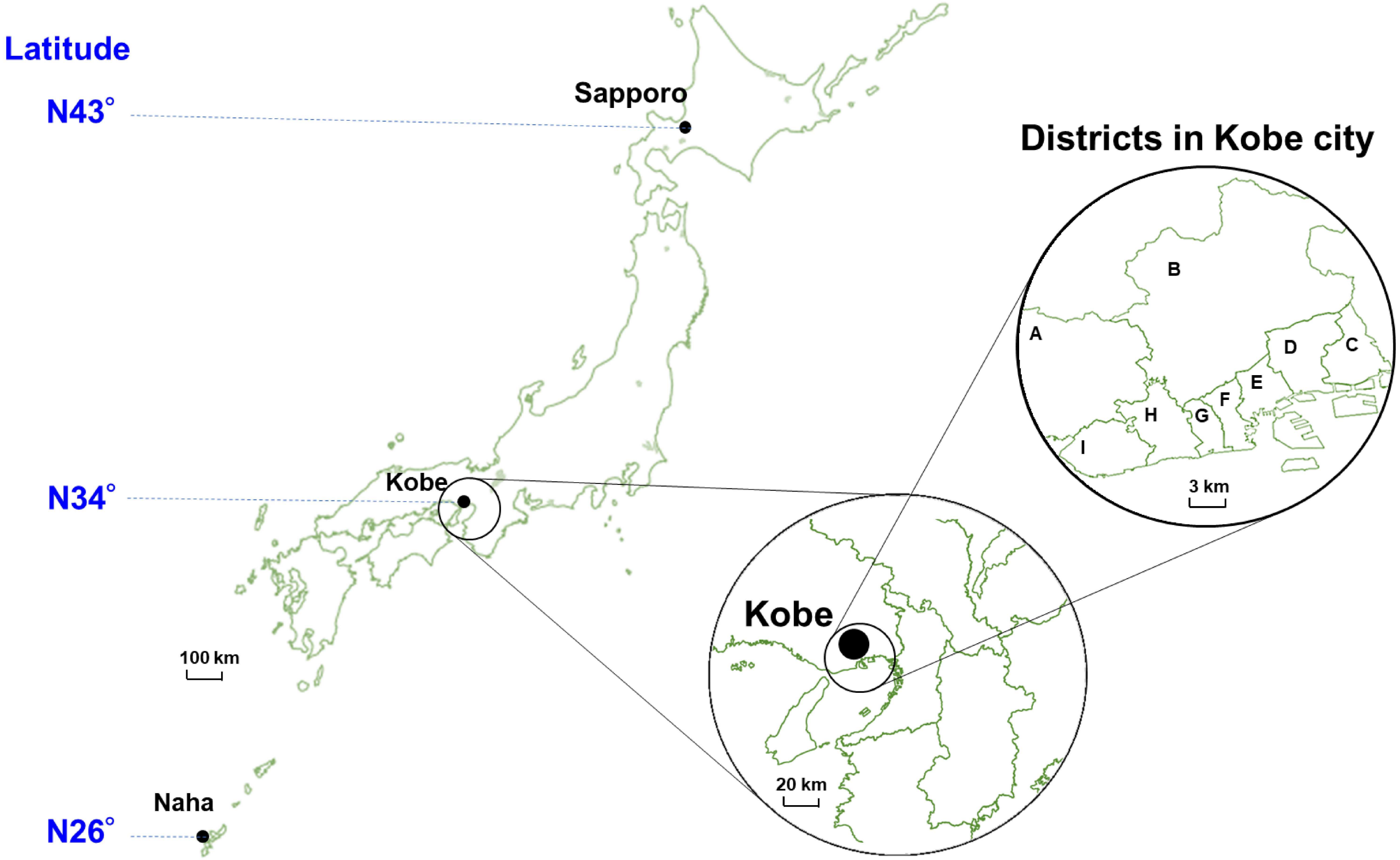

Supplementary Figure 2. Incidence of OHCA by meteorological condition

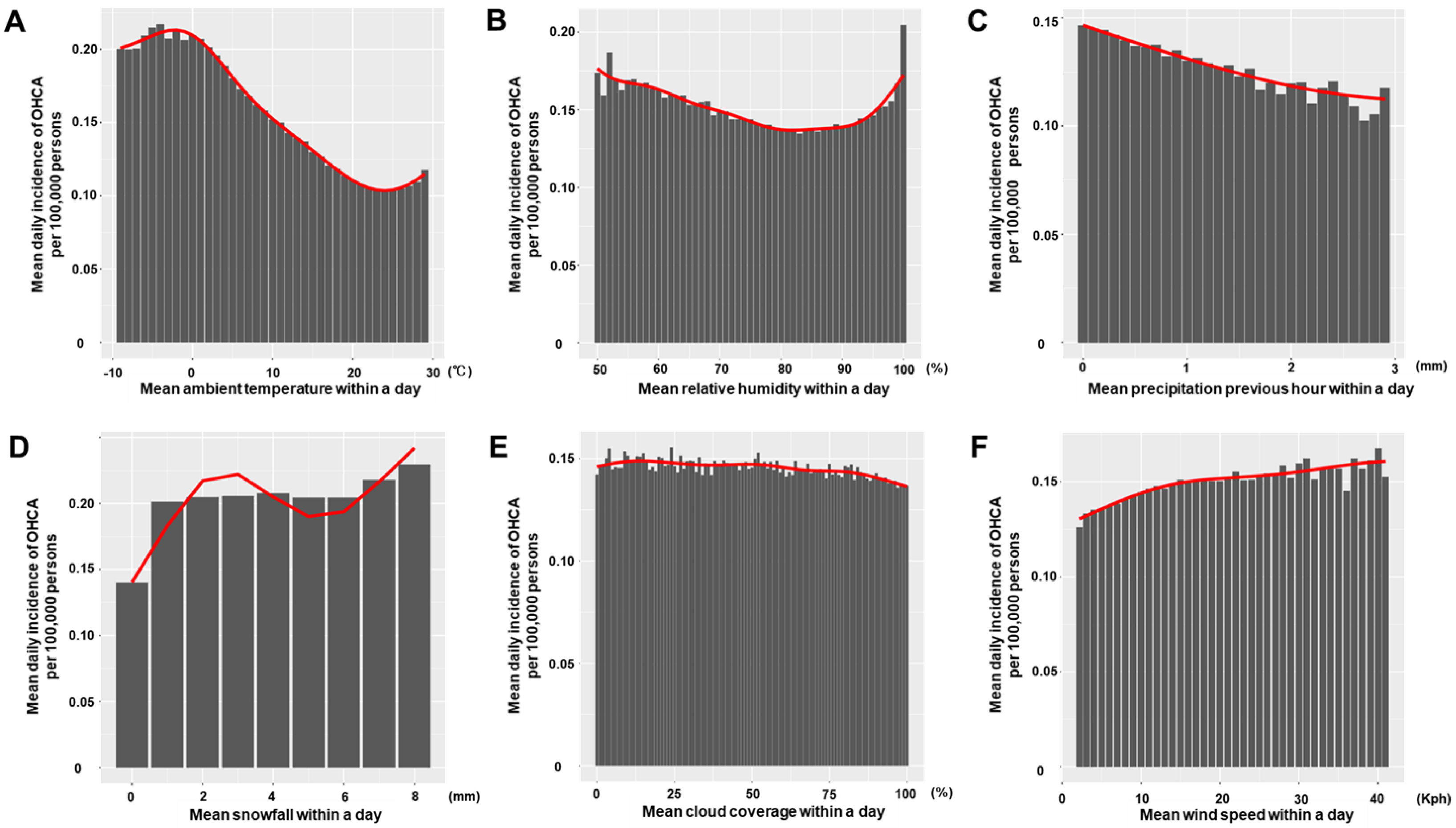

Bars indicate the mean daily incidence of OHCA per 100,000 persons.

OHCA denotes out-of-hospital cardiac arrest.

Nakashima T, *et al. Heart* 2021; 107:1084–1091. doi: 10.1136/heartjnl-2020-318726

Figure 3. Correlations between observed and predicted numbers of out-of-hospital cardiac arrests

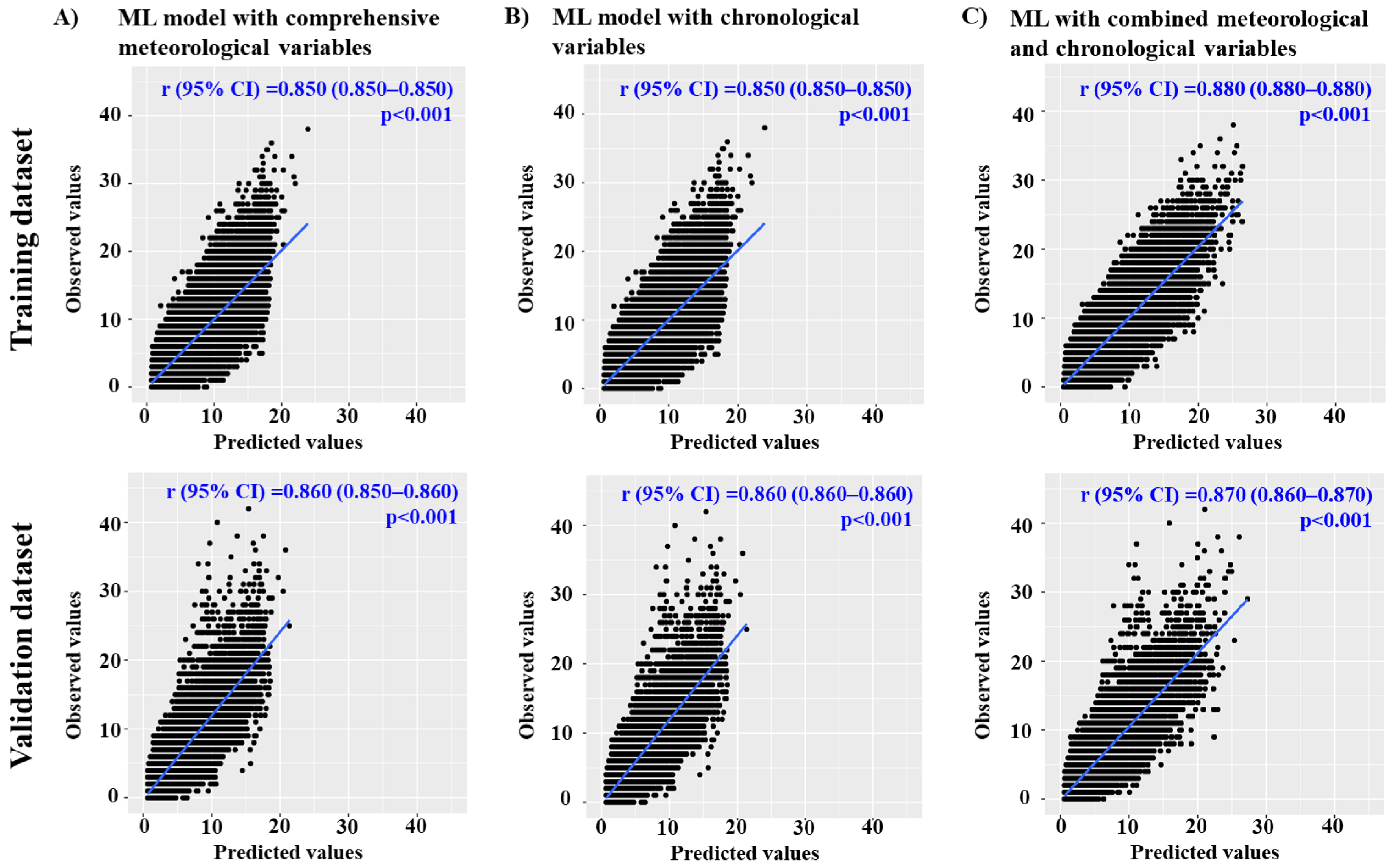

Blue lines are regression lines between observed and predicted values.

CI denotes confidence interval; ML, machine learning; r, correlation coefficient.

References

1. Jacobs I, Nadkarni V, Bahr J, et al. Cardiac arrest and cardiopulmonary resuscitation outcome reports: update and simplification of the Utstein templates for resuscitation registries: a statement for healthcare professionals from a task force of the International Liaison Committee on Resuscitation (American Heart Association, European Resuscitation Council, Australian Resuscitation Council, New Zealand Resuscitation Council, Heart and Stroke Foundation of Canada, InterAmerican Heart Foundation, Resuscitation Councils of Southern Africa). *Circulation.* 2004;110(21):3385-3397.
2. Kitamura T, Iwami T, Kawamura T, et al. Nationwide public-access defibrillation in Japan. *N Engl J Med.* 2010;362(11):994-1004.
3. Kitamura T, Kiyohara K, Sakai T, et al. Public-Access Defibrillation and Out-of-Hospital Cardiac Arrest in Japan. *N Engl J Med.* 2016;375(17):1649-1659.
4. Hasegawa M, Abe T, Nagata T, Onozuka D, Hagihara A. The number of prehospital defibrillation shocks and 1-month survival in patients with out-of-hospital cardiac arrest. *Scand J Trauma Resusc Emerg Med.* 2015;23:34.
5. Nagao K, Nonogi H, Yonemoto N, et al. Duration of Prehospital Resuscitation Efforts After Out-of-Hospital Cardiac Arrest. *Circulation.* 2016;133(14):1386-1396.
6. Japanese Circulation Society Resuscitation Science Study G. Chest-compression-only bystander cardiopulmonary resuscitation in the 30:2 compression-to-ventilation ratio era. Nationwide observational study. *Circ J.* 2013;77(11):2742-2750.
7. Izawa J, Komukai S, Gibo K, et al. Pre-hospital advanced airway management for adults with out-of-hospital cardiac arrest: nationwide cohort study. *BMJ.* 2019;364:l430.
8. Nakashima T, Noguchi T, Tahara Y, et al. Public-access defibrillation and neurological

outcomes in patients with out-of-hospital cardiac arrest in Japan: a population-based cohort study. *Lancet.* 2020;394(10216):2255-2262.
